# Integrating multi-omics approaches reveals metabolic reprogramming and identifies PDHX as a candidate node in triptolide-treated non-small Cell lung cancer

**DOI:** 10.3389/fphar.2026.1785207

**Published:** 2026-04-21

**Authors:** Quancheng Yang, Siqi Chen, Mengjia Sun, Xuejia Zhai, Yi Lv

**Affiliations:** 1 Department of Pharmacy, Union Hospital, Tongji Medical College, Huazhong University of Science and Technology, Wuhan, China; 2 Hubei Province Clinical Research Center for Precision Medicine for Critical Illness, Wuhan, China; 3 Department of Pharmacy, Tongji Hospital, Tongji Medical College, Huazhong University of Science and Technology, Wuhan, China

**Keywords:** chemoresistance, metabolic reprogramming, non-small cell lung cancer (NSCLC), oxidative phosphorylation (OXPHOS), pyruvate dehydrogenase complex X (PDHX), triptolide (TPL)

## Abstract

**Introduction:**

Metabolic reprogramming is a central driver of malignant progression in non-small cell lung cancer (NSCLC). However, conventional targeted therapies face significant limitations due to drug resistance and narrow therapeutic windows. Triptolide, a natural tricyclic diterpenoid derived from Tripterygium wilfordii, exhibits potent antitumor activity, yet its precise mechanisms for modulating metabolic reprogramming in NSCLC remain elusive.

**Methods:**

Using NSCLC cell models, we assessed TPL effects on proliferation, migration, and mitochondrial function *via* CCK-8, Transwell, ROS, and MMP assays. *In vivo* efficacy was evaluated in xenograft models. Untargeted metabolomics identified metabolic alterations, while DARTS proteomics screened for potential TPL-interacting proteins.

**Results:**

TPL significantly inhibited NSCLC cell proliferation and induced metabolic alterations characterized by glycolytic suppression (HK2 downregulation) and concurrent disruption of mitochondrial oxidative phosphorylation (OXPHOS)-associated proteins. Metabolomics revealed systemic metabolic shifts, with pyruvate metabolism and glutathione pathways being most significantly altered. Mechanistically, multi-omics analysis identified PDHX as a key node within a broader metabolic network disrupted by TPL, associated with glycolytic suppression (*via* HK2 degradation) and mitochondrial dysfunction.

**Conclusion:**

These findings suggest that TPL exerts antitumor effects in NSCLC by disrupting both glycolysis and mitochondrial function, with PDHX identified as a candidate mediator. Further studies are warranted to explore its therapeutic potential.

## 1 Introduction

For a long period of time, the high incidence and mortality of lung cancer have consistently been the major cause of death among cancer patients (Sung et al., 2021). Non-small cell lung cancer (NSCLC) represents the most prevalent subtype of lung cancer, accounting for approximately 85% of all cases. The existing therapeutic approaches encompass surgical resection, chemotherapy, radiotherapy, targeted therapy, and immunotherapy. The advancement of targeted therapy inhibitors has constantly enhanced the management of NSCLC(2). Nevertheless, fewer than 25% of patients can benefit from targeted therapy, and drug resistance emerges almost universally during the treatment process (Doroshow and Herbst, 2018). Therefore, expanding treatment strategies and developing effective drugs for lung cancer remain of crucial importance. Metabolic reprogramming is one of the characteristic hallmarks of tumors (Lian et al., 2022). These metabolic alterations support the rapid proliferation of tumor cells, their survival in adverse microenvironments, and their tolerance to treatment. Although the Warburg effect is regarded as one of the features of cancer metabolism (that is, tumor cells preferentially depend on glycolysis for energy generation even in the presence of oxygen), the central role of mitochondria in cellular energy production, oxidative phosphorylation, and cancer has been receiving escalating attention (Wu et al., 2023). Targeting mitochondrial metabolism holds the potential to be developed as an effective strategy for cancer therapy (Sainero-Alcolado et al., 2022; Liberti and Locasale, 2016; Wang and Patti, 2023).

Despite these advances, single-omics approaches often fail to capture the complexity of metabolic networks, this underscores the need for multi-omics strategies to dissect metabolic vulnerabilities holistically. Multi-omics integration strategies (transcriptomics, metabolomics, proteomics) provide key tools for the systematic analysis of metabolic reprogramming in lung cancer (Yan et al., 2025). Transcriptomics revealed abnormally high expression of glycolysis-related genes (such as HK2 and LDHA) and downregulation of mitochondrial oxidative phosphorylation (OXPHOS) pathway in lung cancer cells, directly reflecting the metabolic characteristics of the Warburg effect (Wang et al., 2016; Li et al., 2020). Metabolomics further quantifies the accumulation of glycolytic intermediates such as lactic acid and pyruvate, and the metabolic imbalances of alternative energy sources such as glutamine and lipids, while proteomics identifies changes in the activity of metabolic enzymes and their regulatory networks (Qi et al., 2021; Alvarez et al., 2023). Together, these techniques reveal the molecular mechanisms that support the rapid proliferation and survival of lung cancer cells by remodeling metabolic pathways, such as enhancing glycolysis and inhibiting OXPHOS (Chen et al., 2023). In addition, metabolite exchange in the tumor microenvironment (TME), such as lactate shuttle and glutamine competition, has been confirmed by multi-omics analysis to reshape the immunosuppressant microenvironment and drive therapeutic resistance, providing a theoretical basis for targeting metabolic vulnerability.

Triptolide, a tricyclic diterpene lactone extracted from Tripterygium wilfordii, has garnered widespread attention due to its potent anti-cancer characteristics (Gao et al., 2021). Existing studies have indicated that triptolide acts on multiple tumors such as pancreatic cancer and colon cancer through mechanisms like inducing apoptosis, inhibiting proliferation, and disrupting the tumor microenvironment (AbdulHussein et al., 2023). Emerging evidence reveals that triptolide can also affect tumor metabolism by suppressing hexokinase HK-2 and glycolysis (Cai et al., 2021). To clarify the biochemical mechanisms by which TPL inhibits cell proliferation and metastasis and triggers apoptosis in lung cancer cells from a metabolic perspective, we propose to adopt non-targeted metabolomics combined with Darts proteomics analysis to investigate the systematic impacts of TPL on non-small cell lung cancer cells. In this context, we investigated the molecular targets of triptolide, a natural compound with promising anticancer activity. Non-targeted metabolomics disclosed that TPL markedly interfered with pyruvate metabolism and the tricarboxylic acid cycle (TCA). Through DARTS screening, multiple key metabolic enzymes were identified as potential interacting proteins of TPL, including the mitochondrial pyruvate dehydrogenase complex subunit PDHX. Our findings suggest that triptolide may exert its antitumor effects by targeting PDHX, thus disrupting mitochondrial metabolism and inhibiting tumor cell growth. Combining DARTS with UPLC-Q-TOF-MS-based metabolomics enables a comprehensive analysis of the direct molecular interactions of compounds and their downstream metabolic effects. This combined strategy has significant advantages in revealing the mechanism of action of traditional Chinese medicine extracts and offers important potential for the development of anti-cancer drugs.

## 2 Materials and methods

### 2.1 Chemicals and reagents

Triptolide (10 mM in DMSO) was purchased from MedChemExpress. Formic acid (LC/MS grade, 99% purity) was purchased from Aladdin (Shanghai, China). LC/MS grade methanol and acetonitrile were obtained from Thermo Fisher Scientific. Ultrapure water from an ultrapure water meter (Millipore, USA) was used throughout the study. Cell culture reagents (RPMI-1640 medium, Trypsin, Phosphate Buffered Saline, Penicillin/streptomycin mixture) were purchased from Pricella (Wuhan, China). CCK-8 test kit and crystal violet dye were obtained from Beyotime (Shanghai, China). Kits for ROS and JC-1 were purchased from Servicebio (Wuhan, China).

### 2.2 Cell lines

NCI-H1299 (CL-0165) and NCI-H460 [H460] (CL-0299) were kindly provided by Wuhan Pricella Biotechnology Co.,Ltd. The cells were cultured in RPMI-1640 medium with 10% fetal bovine serum(FBS) and 1% penicillin streptomycin mixture. Cell culture conditions: air, 95%; CO_2_, 5%, 37 °C. Complete RPMI-1640 medium containing different concentrations of TPL was prepared by diluting the stock TPL solution (10 mM in DMSO) in culture medium. The tumor cells were then cultured in a TPL-treated medium.

### 2.3 Cell viability assay

The 96-well plate was inoculated with 100 µL of cell suspension. Then, triptolide intervention cells with different concentrations (eight concentration gradients from 0 nM to 100 nM) were added to the culture plate in the form of liquid exchange, and incubated in the incubator for 48 h, with three multiple pores for each concentration. Cell viability was measured using the CCK-8 kit according to the manufacturer’s instructions.

### 2.4 Cell migration assay

The cells were counted and inoculated into the 6-well plate at a concentration of about 5 × 10^4^ cells/mL until the cells were fully grown and scratched. Complete medium containing different concentrations of triptolide (0 nM, 2.5 nM, 5 nM, 10 nM) was added to treat the cells, and the migration of tumor cells was recorded by photographing every 6 h.

The cells were re-suspended in serum-free medium and then inoculated into the Transwell chamber, where about 200 µL of liquid was placed, and 500 µL of complete medium containing different concentrations of TPL was added into the chamber under the 24-well plate. The cells were cultured in the incubator for 48 h, then fixed, stained, and photographed.

### 2.5 Colony formation assay

Each concentration group was inoculated with about 500 cells/Wells in the 6-well plate culture plate, fully blown into cell suspension in the form of single cells. The cells were cultured in the cell incubator for about 2 weeks, and the culture vial was replaced with complete media containing different concentrations of triptolide (0 nM, 2.5 nM, 5 nM, 10 nM) every 2–3 days, and the cells could be fixed if the number of single clone cells reached about 50. The cells were fixed with paraformaldehyde for 15 min per well, and stained with 0.1% crystal violet solution for 10–20min. The cells were washed with PBS several times and the results were recorded with photos.

### 2.6 Cell reactive oxygen species detection

Cells in the logarithmic growth phase were harvested, resuspended in complete medium, and counted. After serial dilution, approximately 5 × 10^3^ cells/mL were seeded into 35 mm confocal dishes and cultured for 24 h to allow attachment. When cell confluence reached 50%–70%, the medium was replaced with 250 µL of complete medium containing different concentrations of triptolide (0, 2.5, 5, or 10 nM) for 48 h. For staining, the medium was aspirated and cells were washed once or twice with PBS. Following the manufacturer’s protocol, an appropriate volume of DCFH-DA working solution was added, and cells were incubated at 37 °C in a CO_2_ incubator for 30 min in the dark. After removal of the DCFH-DA solution, cells were washed 2–3 times with PBS to remove excess probe, and finally covered with PBS. Detection was performed using a confocal microscope with excitation at 488 nm and emission at 525 nm (FITC channel). Images were captured and recorded for analysis.

### 2.7 Cell metabolome assay

NCI-H1299 cells and NCI-H460 cells were divided into low (2.5 nM), medium (5 nM), high (10 nM) TPL concentration treatment group and Control group (complete culture medium without TPL), and the cells in each group were treated with drugs or blank media for 72 h. After washing twice with PBS in ice water, fix with 1 mL 80% ice methanol. Scrape the cells from the plate, add 0.5 mm beads, and treat the cells by grinding and shaking them (treat them on ice). After centrifugation, the supernatant was collected and mixed with 800 μL acetonitrile on ice for deproteinization. The supernatant is then collected and rotated under nitrogen at room temperature. The samples were re-suspended in 100 μL distilled water, added with an internal standard (1 mg/mL LPC(12:0)) of 5 μL, mixed and transferred into injection vials. 5 μL was used for metabolome analysis based on LC-MS.

Metabolomics analysis was performed using UHPLC-TOF/MS. The chemical separation of metabolomics was based on the obtained UPLC HSS T3 (2.1 × 100mm, 1.7 μm) column. The mobile phase A is water containing 0.1% formic acid, and B is acetonitrile containing 0.1% formic acid. The injection volume is set to 5 μL. Chromatographic separation conditions: column temperature was 40 °C; Flow rate 0.3 mL/min; Mobile phase composition A: water +0.1% formic acid, B: acetonitrile +0.1% formic acid; Automatic injector temperature 4 °C. Metabolomics analysis was performed using six biological replicates (n = 6) per treatment group. Quality control (QC) samples were used to optimize the conditions. QC samples were prepared with 10 μL of each sample, and QC samples were added every five sample intervals to evaluate the stability of the sequence.

### 2.8 Cell darts assay

NCI-H460 cells were lysed in WB/IP lysis buffer containing 1× protease inhibitor cocktail at 4 °C for 2 h with vortexing every 30 min. The lysate was centrifuged at 13,000 rpm for 20 min at 4 °C, and the supernatant was collected. Protein concentration was determined using the BCA assay. For the DARTS assay, 200 µL of cell lysate was incubated with either 2 µL DMSO (control) or 2 µL triptolide (final concentration 100 µM) for 1 h at room temperature. Pronase was then added at an enzyme-to-protein ratio of 1:300 and incubated for 30 min. Protease activity was terminated by adding 20× protease inhibitor cocktail, and samples were denatured for SDS-PAGE analysis. Protein bands of interest were excised from the gel and destained. Reduction was performed with dithiothreitol (DTT) at 57 °C for 60 min, followed by alkylation with iodoacetamide in the dark for 30 min. After dehydration with acetonitrile, proteins were digested overnight with trypsin at 37 °C. The resulting peptides were extracted, desalted using C18 Ziptips, and dried by vacuum centrifugation. Peptides were reconstituted in 0.1% formic acid and analyzed using an EASY-nLC 1200 system coupled with a Q-Exactive HF mass spectrometer (Thermo Scientific). Peptides were separated on a C18 analytical column (75 μm × 15 cm, 1.9 µm particles) at a flow rate of 300 nL/min. MS data were acquired in data-dependent acquisition mode with a full scan range of 350–1500 m/z at 60,000 resolution. The top 20 precursor ions were selected for higher-energy collisional dissociation (HCD) fragmentation with a normalized collision energy of 25%. Raw MS data were processed using Proteome Discoverer software (version 2.4, Thermo Scientific).

### 2.9 Animal treatment

**FIGURE 1:**
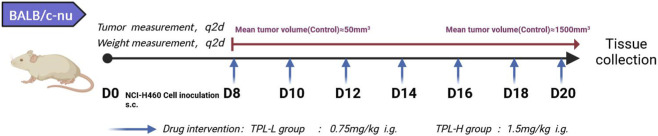
Schematic representation of the tumor-formation animal experiment.

Five-week-old male BALB/c nude mice were purchased from Hubei Beiente Biotechnology and housed under SPF conditions (23 °C ± 2 °C, 40% humidity) with free access to food and water. All animal procedures were approved by the Ethics Committee of Huazhong University of Science and Technology. NCI-H460 cells (1 × 10^7^ cells/mL in PBS) were injected subcutaneously into the left axilla of each mouse (Figure 1). Tumor volume was calculated as V = (A × B^2^)/2, where A is the long diameter and B is the short diameter. When tumors reached 50–100 mm^3^ (approximately 7 days), mice were randomly divided into six groups (n = 8 per group): Control, Huobahuagen Tablets (HBHGP), TPL-L, TPL-H, gefitinib (GF), and GF + TPL. Mice were sacrificed when control tumors reached 1000–1500 mm^3^. Blood, tumors, and major organs were collected for analysis. The study was designed to evaluate dose-dependent efficacy of TPL, benchmark against gefitinib, explore combination effects, and assess the TPL-containing formulation Huobahuagen Tablets. Tumor measurements were performed by an investigator blinded to group assignment.

Tissue samples were lysed in RIPA buffer containing protease inhibitors on ice. The lysate was centrifuged at 12,000 rpm for 5 min at 4 °C, and the supernatant was collected. Protein concentration was determined using the BCA assay. Equal amounts of protein (20 μg per lane) were separated by SDS-PAGE and transferred onto PVDF membranes. Membranes were blocked with 5% non-fat milk for 1 h at room temperature, then incubated with primary antibodies overnight at 4 °C. After washing with TBST, membranes were incubated with HRP-conjugated secondary antibodies for 30 min at room temperature. Protein bands were visualized using enhanced chemiluminescence (ECL) and quantified using AlphaEaseFC software (Figure 1).

### 2.10 Statistical analysis

All experiments were performed with at least three independent biological replicates unless otherwise specified. For cell-based assays, each biological replicate consisted of cells from a separate passage or culture preparation. Technical replicates (e.g., multiple wells per condition within the same experiment) were averaged and treated as a single data point per biological replicate. Data are presented as mean ± standard deviation (SD) from biological replicates.

Statistical analyses were performed using GraphPad Prism software (version 8.0, GraphPad Software, San Diego, CA, USA). For comparisons between two groups, unpaired two-tailed Student's t-test was used. For multiple group comparisons, one-way ANOVA followed by Dunnett’s or Tukey’s *post hoc* test was applied. For metabolomics data, p-values from univariate analysis (ANOVA) were adjusted for multiple comparisons using the Benjamini–Hochberg false discovery rate (FDR) method. Metabolites with VIP > 1 and adjusted p < 0.05 were considered significantly altered. For DARTS proteomics data, p-values from unpaired t-tests were similarly adjusted using the Benjamini–Hochberg FDR method, and proteins with fold change ≥1.2 or ≤0.833 and adjusted p < 0.05 were considered significantly differentially expressed. Statistical significance was set at p < 0.05 (*), p < 0.01 (**), and p < 0.001 (***). Exact p-values are provided for all comparisons where p ≥ 0.001; p < 0.001 is reported as such. Effect sizes are reported as percentage change relative to control or as mean differences with 95% confidence intervals where applicable.

## 3 Results

### 3.1 Triptolide inhibits the proliferation and the migration of human non-small cell lung cancer cells

**FIGURE 2:**
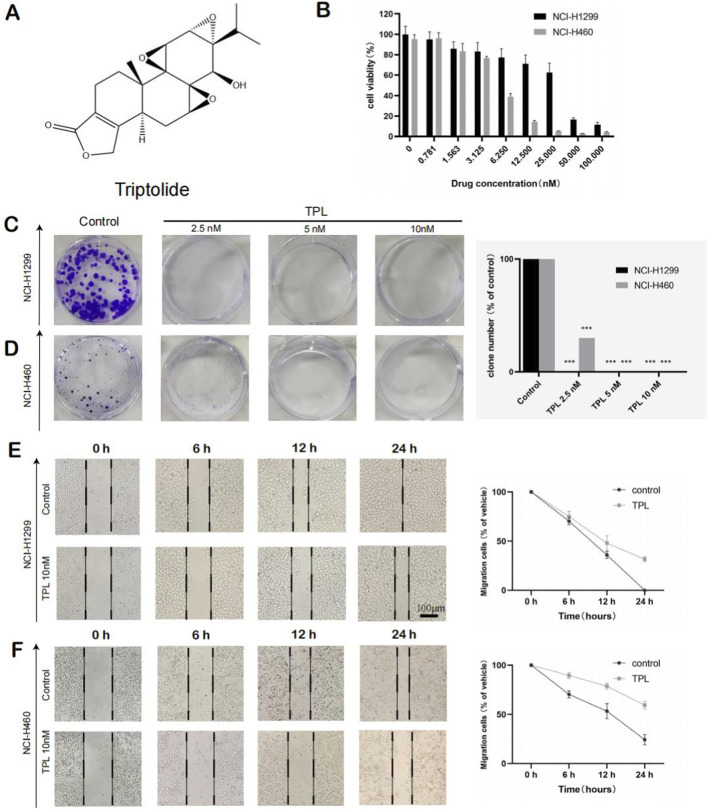
Anti-proliferation effect of Triptolide on NSCLC cell lines. **(A)** Chemical structure of Triptolide. **(B)** The Effect of TPL on proliferation ability of NCI-H1299 and NCI-H460 detected by CCK-8 experiment (Data represent from three independent experiments (n = 3 biological replicates), with three technical replicates per condition per experiment). **(C)** The effect of TPL on the proliferation ability of NCI-H1299 cell line was examined by clonal formation assay (***p < 0.001, ***p vs. control group), Representative images from three independent experiments (n = 3 biological replicates). **(D)** The effect of TPL on the proliferation ability of NCI-H460 cell line was examined by clonal formation assay. **(E)** The effect of TPL on the migration ability of NCI-H1299 was detected by scratch test. Data represent mean from three independent experiments (n = 3 biological replicates), with at least five fields of view analyzed per condition per experiment. **(F)** The effect of TPL on the migration ability of NCI-H460 was detected by scratch test (***p < 0.001, ***p vs. control group).

The structure of triptolide is shown in Figure 2A. In order to explore the effect of triptolide on the proliferation of non-small cell lung cancer cells, the non-small cell lung cancer cell lines NCI-H1299 and NCI-H460 were selected as the research objects in this study, and CCK-8 method was used to detect the intervention ability of different concentrations of TPL on cell proliferation. The experimental results were shown in Figure 2B. As the dose of TPL was gradually increased, the cell proliferation ability of NCI-H1299 and NCI-H460 was gradually inhibited. The half inhibitory concentrations of the 2 cell lines were shown in Supplementary Figure S1. Four TPL concentrations were set for subsequent experiments according to the IC50 value, which were 0 nM, 2.5 nM, 5 nM and 10 nM, respectively. The clone formation experiment results showed that the number of clones in the TPL administration group was significantly reduced compared with the control group, and Quantitative analysis confirmed a dose-dependent inhibition (e.g., for H460 cells: 10 nM TPL reduced colony formation to 18.4% ± 5.1% of control, *p* < 0.001) (Figures 2C–D).

**FIGURE 3:**
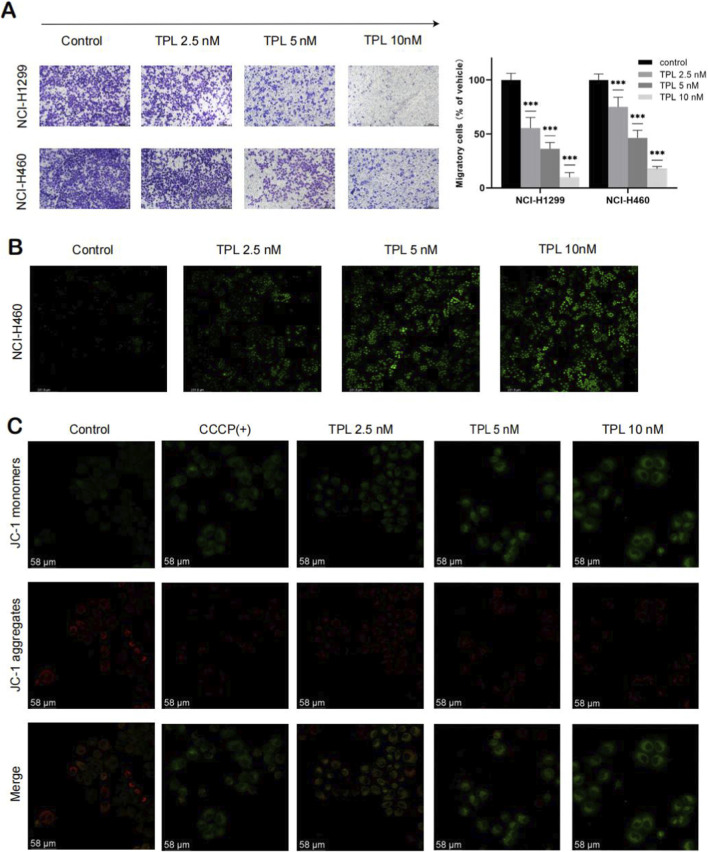
**(A)** Transwell experiment was conducted to detect the effect of TPL on the migration ability of NCI-H1299 and NCI-H460 (***p < 0.001, ***p vs. Control group). **(B)** The effect of TPL on the production of reactive oxygen species (ROS) in NCI-H460 was detected by ROS assay. **(C)** The effect of TPL on the generation of mitochondrial membrane potential in NCI-H460 was detected by JC-1 assay (CCCP: positive control group). Representative confocal images from three independent experiments (n = 3 biological replicates). Quantification of fluorescence intensity was performed using ImageJ with at least five fields of view per experiment.

Next, the scratch wound healing test and Transwell assay were used to evaluate whether triptolide affected the migration ability of NCI-H1299 and NCI-H460 cells. The results of the cell scratch test showed that compared with the control group, the cell migration rate and scratch healing rate of the TPL administration group were significantly slowed down as shown in Figure 2E-F and Figure 3A. For instance, the relative wound closure of H460 cells at 24h was 67.3% ± 4.2% in the control group *versus* 23.7% ± 2.8% in the 10 nM TPL group (mean difference: 43.6%, 95% CI: 39.2%–48.0%, p < 0.001). Compared with the control group, TPL treatment significantly reduced the number of migrating cells, with approximately 75% reduction at 10 nM TPL (mean reduction: 74.8%, 95% CI: 68.5%–81.1%, p < 0.001). Triptolide inhibited the migration of non-small cell lung cancer cells in a dose-dependent manner.

### 3.2 Triptolide alters the metabolomic profile of NSCLC cells

**FIGURE 4:**
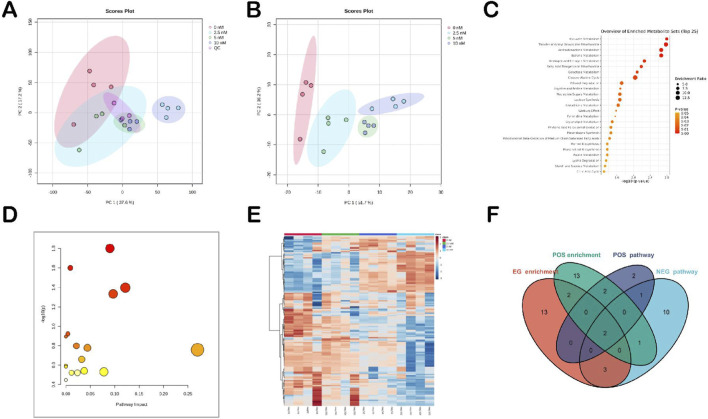
Metabolomics analysis of TPL’s anti-NSCLC effect. **(A,B)** PCA and PLS-DA score plots for NCI-H460 cells, respectively (n = 6 biological replicates per group). **(C)** SMPDB analysis of common differential metabolites; **(D)** KEGG enrichment analysis of common differential metabolites; **(E)** Heat map of differential metabolites (VIP > 1, ANOVA p < 0.05, with the Benjamini–Hochberg FDR correction for multiple comparisons); **(F)** Venn diagrams of common pathways for differential metabolism under different ion modes. Data were analyzed using Progenesis®QI, and pathways with p < 0.05 after FDR correction were considered significantly enriched.

As illustrated in Figures 4A,B, the initial PCA plot demonstrated the metabolic differences between the TPL-treated group and the Control group (i.e., the 0 nM group). Notably, there was a distinct separation between the samples of the TPL-treated group and the Control group, particularly within the high-concentration treatment subgroup. These findings indicate that TPL intervention significantly alters the metabolic profile of non-small cell lung cancer. The criteria for differential metabolite screening were set as VIP > 1 and Anova p-value ≤ 0.05. A multi-group comparison model (Control vs. 2.5 nM vs. 5 nM vs. 10 nM) was established, resulting in the identification of 45 differential metabolites. Heatmap was generated based on the peak intensity of each metabolite as relative content to visualize the distribution of differential metabolites across groups (Figure 4E). According to the VIP value and Anova p-value, potential metabolic markers associated with tumor metabolic activity include the following: Pyroglutamic acid, Malic acid, NADP, L-Proline, S-Lactoylglutathione, NADH, Thymidine, N,N′-Bis(gamma-glutamyl)cystine, N-Acetyl-L-methionine, N-Acetylserine. The results of KEGG pathway analysis and SMPDB enrichment analysis were combined. Pyruvate metabolism and glutathione metabolismwere the common metabolic pathways in the two groups (Figure 4F), suggesting their potential involvement in TPL-mediated anti-NSCLC effects. Metabolomics analysis revealed significant changes in metabolites associated with glycolysis (e.g., pyruvate) and the TCA cycle, suggesting perturbations in these pathways.

### 3.3 Triptolide induces ROS accumulation and mitochondrial dysfunction in NCI-H460 cells

We detected changes in reactive oxygen species (ROS) and mitochondrial membrane potential (MMP) in tumor cells. The results showed that in NCI-H460 cells, the ROS levels in the drug-treated group were significantly higher compared to the control group (Figure 3B) Concurrently, the MMP, measured by JC-1 red/green fluorescence ratio, showed a dose-dependent decrease (Figure 3C), with a ∼40% reduction at 10 nM TPL (p < 0.001), indicating mitochondrial depolarization.

### 3.4 Triptolide treatment significantly downregulates key proteins involved in metabolic reprogramming and cell survival

**FIGURE 5:**
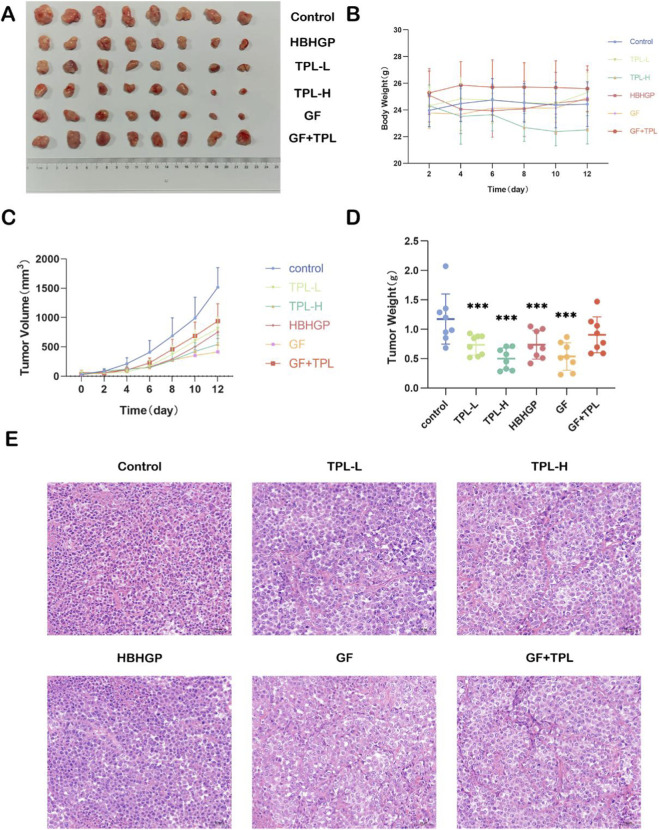
Effects of TPL and gefitinib alone or in combination on the growth of non-small cell lung cancer in tumor-bearing mice (n = 8 per group) **(A)** stripped nude mouse tumors. **(B)** The change of body weight of nude mice with the time of administration. **(C)** Changes in tumor volume with time of administration. **(D)** Tumor weight distribution (***p < 0.001, ***p vs. control group). **(E)** HE staining results of tumor tissue after triptolide administration.

**FIGURE 6:**
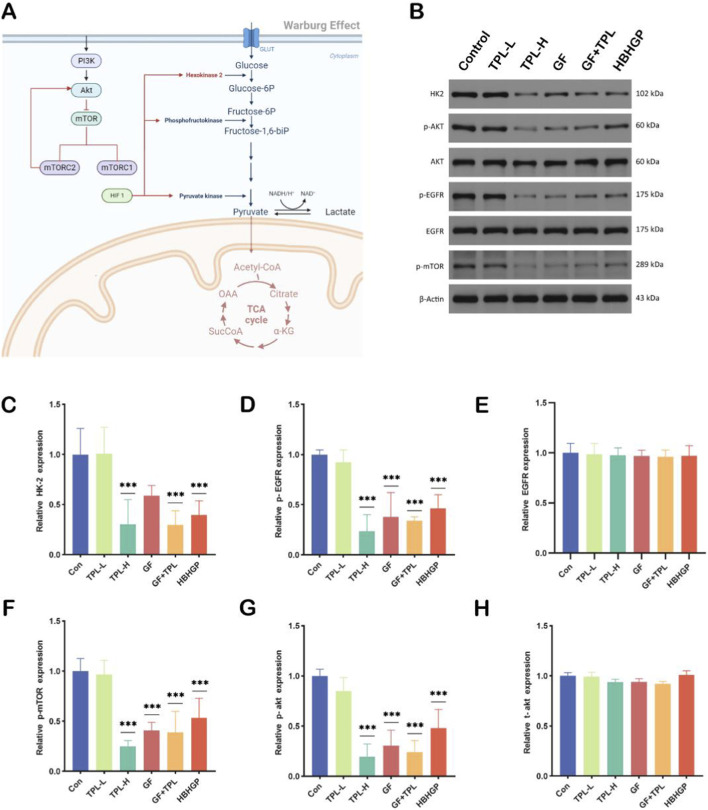
**(A)** Pathways related to glycolytic proteins. **(B)** Expression of glycolytic-related proteins. **(C)** HK 2 protein expression was evaluated by immunoblotting in NCI-H460. **(D)** p-EGFR protein expression was evaluated by immunoblotting in NCI-H460. **(E)** EGFR protein expression was evaluated by immunoblotting in NCI-H460. **(F)** p-mTOR protein expression was evaluated by immunoblotting in NCI-H460. **(G)** p-akt protein expression was evaluated by immunoblotting in NCI-H460. **(H)** t-akt protein in expression was evaluated by immunoblotting in NCI-H460. (***p < 0.001, ***p vs. control group). Representative blots from three independent experiments (n = 3 biological replicates).

Metabolomics findings unveiled that triptolide influences the energy metabolism process and exerts anti-tumor actions at the cellular level (Figure 4). Hence, we subsequently adopted the tumor-bearing mouse model to assess the *in vivo* anti-tumor efficacy of triptolide. In this research, gefitinib was employed as the positive control drug, and the Huoba Huagen Tablets are a traditional Chinese medicine formulation with triptolide as the principal component. As illustrated in Figures 5C,D, final tumor volume in the control group was 1395 ± 210 mm^3^, while the TPL-H group showed a significant reduction to 520 ± 95 mm^3^ (mean difference: 875 mm^3^, 95% CI: 665–1085 mm^3^, p < 0.001). Mean tumor weight was 1.3 ± 0.2 g in the control group *versus* 0.5 ± 0.1 g in the TPL-H group (mean difference: 0.8 g, 95% CI: 0.6–1.0 g, p < 0.001).The experimental outcomes demonstrated that both the TPL-L group and the TPL-H group could conspicuously inhibit the tumor growth in nude mice (vs. the Control group, *p* < 0.001), and a dose-dependent effect existed. The higher the concentration of triptolide was, the more pronounced the tumor inhibitory effect was (TPL-L vs. TPL-H, p = 0.023). In this study, gefitinib was adopted as the positive control group. The therapeutic efficacy of the low-dose TPL group was inferior to that of the GF group (TPL-L: 820 ± 110 mm^3^ vs. GF: 580 ± 85 mm^3^, p = 0.017), while the therapeutic efficacy of the high-dose TPL group was comparable to that of the gefitinib group (TPL-H: 520 ± 95 mm^3^ vs. GF: 580 ± 85 mm^3^, p = 0.342). The HBHGP group administered 600 mg/kg of Huobahuagen Tablets to nude mice by gavage, and the amount of triptolide contained therein was far lower than that of the TPL-L group; However, the HBHGP group showed tumor inhibitory effect similar to that of the TPL-L group (HBHGP: 790 ± 105 mm^3^ vs. TPL-L: 820 ± 110 mm^3^, p = 0.561). Besides, no overt signs of toxicity (e.g., significant body weight loss or major organ damage on histology were manifested in all the administration groups. As the administration concentration of TPL increased, a dose-dependent enhancement in tumor suppression was observed (Figures 5B–D). Tumor HE staining and alterations in mouse body weight were utilized to evaluate the safety and effectiveness of TPL *in vivo* treatment (Figure 5E). The results of the HE staining experiment indicated that in the Control group, tumor cells were closely arranged, and the nuclei were uniformly circular. In the treatment groups, tumor cells displayed a less compact arrangement with increased intercellular spacing. Compared to the Control group, the treatment groups showed reduced nuclear size, presence of vacuoles, and expanded necrotic areas, consistent with TPL-induced tumor regression. Western blotting analysis revealed a marked reduction in hexokinase 2 (HK2, reduced to nearly35% of control at TPL-H group in tumor, *p* < 0.001), a crucial enzyme in glycolysis, indicating TPL disrupts glycolytic activity (Figures 6B,C). Additionally, TPL inhibits the phosphorylation of Akt (p-Akt) and EGFR (p-EGFR)(TPL-H vs. control, *p* < 0.001), suggesting a blockade of cell survival and proliferation signals (Figures 6F,G). However, total protein levels of Akt remained unchanged, implying that TPL affects the activation rather than the expression (Figures 6G,H). These findings highlight that TPL may inhibit NSCLC cell growth by targeting both metabolic pathways and survival signaling mechanisms.

### 3.5 Direct interaction of triptolide with key proteins involved in metabolic reprogramming and energy metabolism

**FIGURE 7:**
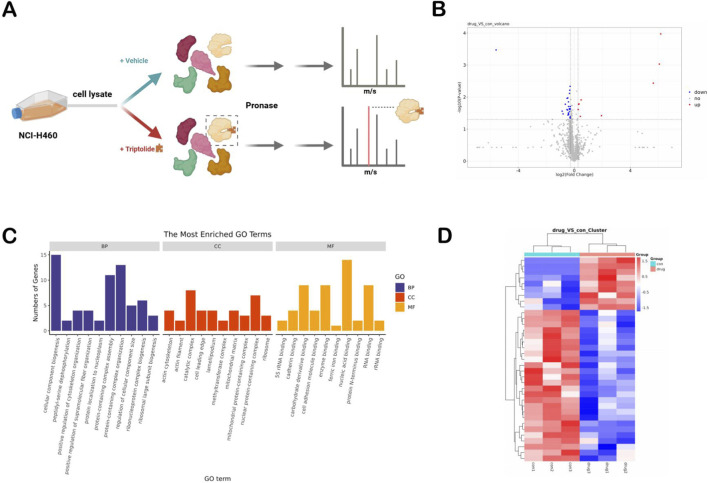
Results of Darts proteomic experiments **(A)** Schematic diagram of the Darts experiment procedure. **(B)** Volcano plots of differential gene expression matrices (TPL vs. Control group n = 3 independent experiments),Proteins with fold change ≥1.2 or ≤0.833 (i.e., 1/1.2) and p < 0.05 (unpaired t-test, with the Benjamini–Hochberg FDR correction for multiple testing) were considered significantly altered. Red dots: upregulated proteins; blue dots: downregulated proteins. **(C)** GO analysis of differential gene expression matrices (p < 0.05 after FDR correction). **(D)** Heatmap of differential gene expression matrices (TPL vs. Control group, nine upregulated, 26 downregulated)identified by DARTS screening). DARTS proteomics was performed using three independent biological replicates (n = 3) per group.

To determine the potential direct targets of TPL in inhibiting NSCLC, the drug affinity responsive target stability (DARTS) was employed to screen the target proteins of TPL (Figure 7A). NCI-H460 cells were utilized as the protein source for the DARTS assay. The DARTS experiment, as an unlabeled target screening approach, possesses high sensitivity and specificity and can minimize potential interferences. Based on the screening conditions (fold change ≥1.2 or ≤0.8333 (i.e., 1/1.2); p-value < 0.05), 35 differentially expressed proteins affected by the intervention of TPL were screened out. Compared with the control group, 9 proteins were upregulated and 26 proteins were downregulated in the drug group (Figures 7B,D). Among them, the differentially expressed proteins such as PDHX (Pyruvate dehydrogenase protein X component, mitochondrial), MRPL28 (39S ribosomal protein L28, mitochondrial), ACAD9 (Complex I assembly factor ACAD9, mitochondrial), and NDUFB8 (NADH dehydrogenase ubiquinone 1 beta subcomplex subunit 8) are highly correlated with mitochondrial function and energy metabolism (Figure 7; Supplementary Table S1). GO enrichment analysis of TPL targets was performed to evaluate their biological functions, encompassing cellular components, molecular functions, and biological processes (Figure 7C). Additionally, KEGG enrichment analysis revealed that TPL may participate in the energy metabolism processes of non-small cell lung cancer (NSCLC) through multiple signaling pathways, including starch and sucrose metabolism (hsa00500) and insulin resistance. These protein alterations indicate that TPL disrupts the metabolic balance of NSCLC cells by multi-target regulation of metabolic pathways.

### 3.6 Joint Pathway Analysis: integration of proteomics and metabolomics

**FIGURE 8:**
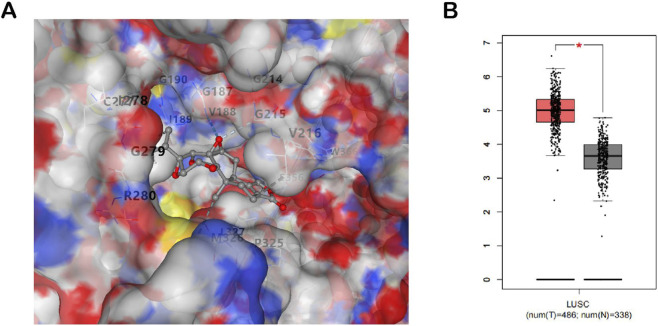
**(A)** Predicted binding mode of TPL and PDHX; **(B)** The differentiated expression of PDHX in LUSC (*p < 0.05, *p vs. control group).

To comprehensively elucidate the integrated effects of drug treatment on cellular metabolism and protein expression, a Joint Pathway Analysis was conducted to integrate proteomics and metabolomics data, identifying several significantly enriched key pathways. The results revealed that glutathione metabolism, pyruvate metabolism, and the TCA cycle were prominently enriched in the combined analysis. These pathways play crucial roles in tumor metabolic reprogramming and the regulation of antioxidant stress responses. In our DARTS experiment, we identified a panel of proteins that potentially interact with triptolide. Among these, PDHX, a key component of the pyruvate dehydrogenase complex, emerged as a top candidate due to its central role in pyruvate metabolism, which aligns with our metabolomics findings. In molecular docking simulations, TPL and PDHX can bind to multiple amino acid residues near the active center through hydrogen bonds, carbon-hydrogen bonds, van der Waals bonds, Pi-Pi stacking, and hydrophobic interactions, with a binding energy of −8.1 kcal/mol (Figure 8A). Gene expression analysis in the TCGA database revealed that PDHX was significantly upregulated in lung cancer tissues (Figure 8B, *p* < 0.05). Additionally, other metabolism-related proteins such as PYGB and TK1 were also identified, suggesting that triptolide may exert its anti-tumor effects by targeting multiple nodes in the metabolic network. In the tricarboxylic acid cycle, the significant changes in key proteins such as NDUFB8 and MPRL28 further support the drug’s interference with mitochondrial oxidative phosphorylation. Further validation of these targets will provide deeper insights into the molecular mechanisms of triptolide and its potential applications in cancer therapy.

## 4 Discussion

The treatment of non-small cell lung cancer (NSCLC) remains a formidable challenge due to the lack of effective targeted therapies and the inherent limitations of chemotherapy, including drug resistance and systemic toxicity. Triptolide (TPL), a natural diterpenoid derived from Tripterygium wilfordii, has demonstrated significant antitumor activity in and modulating energy homeostasis (Cruz-Bermúdez et al., 2019; Eltayeb et al., 2024). While metabolic dysregulation has emerged as a hallmark of cancer progression, with accumulating evidence highlighting its potential to the role of TPL in reshaping metabolic pathways specifically in NSCLC remains underexplored (Avolio et al., 2020; Agarwala et al., 2024).

### 4.1 Multi-omics integration identifies PDHX as a candidate node in TPL-Induced metabolic disruption

The integration of DARTS and untargeted metabolomics provided a powerful synergy for deciphering triptolide’s (TPL) mechanisms, bridging potential direct target identification with systemic metabolic reprogramming. DARTS analysis revealed 35 proteins significantly altered by TPL, including mitochondrial enzymes (PDHX, MRPL28) and glycolytic regulators (HK2). Notably, PDHX upregulation suggested a compensatory response to TPL-induced pyruvate accumulation, while HK2 downregulation directly suppressed glycolysis (Inoue et al., 2021). These findings highlighted TPL’s dual action: blocking glycolysis while perturbing mitochondrial function. Concurrently, metabolomics profiling identified 45 differentially abundant metabolites, with glutathione and pyruvate pathways being most significantly disrupted. Pathway enrichment further linked TPL to glutathione depletion, pyruvate oxidation (PDHX↑), and TCA cycle activation, corroborating DARTS results. While DARTS and metabolomics data converge on PDHX as a high-priority candidate, definitive proof of direct binding will require orthogonal biophysical methods such as surface plasmon resonance or cellular thermal shift assays. Furthermore, functional validation *via* genetic manipulation (e.g., PDHX knockdown or overexpression) is necessary to establish causality. These investigations are underway in our ongoing studies. The technical synergy between DARTS and metabolomics was particularly evident in resolving mechanistic ambiguities: for instance, the downregulation of S-lactoylglutathione—a glyoxalase I product—in metabolomics correlated with PDHX upregulation in DARTS, suggesting TPL disrupts methylglyoxal detoxification by diverting PDHX from glyoxalase activity to pyruvate dehydrogenase function (Luengo et al., 2019). This integrated approach overcame the limitations of single-technology strategies: DARTS alone might have overlooked broader metabolic network dynamics, while metabolomics alone could not distinguish direct targets from downstream effects. In addition, We acknowledge that our conclusions regarding metabolic reprogramming are based on metabolomics and protein expression data rather than direct flux measurements. While these approaches provide a global view of metabolic alterations, they cannot quantify actual pathway activity or distinguish between changes in flux *versus* metabolite abundance. Future studies employing Seahorse analysis to measure oxygen consumption rate (OCR) and extracellular acidification rate (ECAR), along with lactate production, glucose uptake, and ATP partitioning assays, are necessary to confirm the proposed shifts in glycolytic and oxidative metabolism. Isotope tracing would further clarify the direction and magnitude of flux changes.

### 4.2 TPL disrupts glycolysis and mitochondrial function

The findings reveal that triptolide (TPL) exerts its anticancer effects through a dual mechanism involving (Sung et al., 2021): metabolic reprogramming characterized by glycolysis suppression and mitochondrial oxidative phosphorylation (OXPHOS) disruption, and (Riely et al., 2024) mitochondrial dysfunction associated with ROS accumulation and electron transport chain (ETC.) inhibition. At the metabolic level, TPL significantly reduced glycolytic intermediates (e.g., lactate, pyruvate) while increasing OXPHOS-related metabolites (NADH, ATP), suggesting a shift from aerobic glycolysis to mitochondrial respiration—a phenomenon reminiscent of the “reverse Warburg effect.” This metabolic shift was associated with the downregulation of key glycolytic enzymes (HK2, LDHA) and the upregulation of PDHX, a critical component of the pyruvate dehydrogenase complex that facilitates pyruvate entry into the TCA cycle. Notably, the suppression of HK2 (a glycolysis gatekeeper) aligns with previous studies (Cai et al., 2021). Simultaneously, TPL-induced mitochondrial dysfunction, evidenced by reduced membrane potential and downregulated, ETC., proteins (MRPL28, ACAD9, NDUFB8), was associated with increased ROS accumulation. The interplay between ROS and mitochondrial dysfunction may contribute to metabolic stress, although further studies are needed to establish causality. Consistent with these observations, Lin et al. recently highlighted the critical interplay between mitochondrial dysfunction and ROS generation in cellular stress responses, further supporting the notion that TPL-induced metabolic disruption may be mediated through oxidative stress pathways (Lin et al., 2025). The observed association between ROS accumulation and mitochondrial dysfunction mirrors patterns reported in compensatory metabolic adaptation (Xia et al., 2021). While our data demonstrate a clear association between TPL treatment, ROS accumulation, and growth inhibition, we acknowledge that the current study cannot distinguish whether oxidative stress is a primary driver of cell death or a secondary consequence of mitochondrial dysfunction. Elucidating the causal role of ROS will require future investigations using ROS scavengers such as N-acetylcysteine (NAC) in combination with TPL treatment.

### 4.3 *In Vivo* efficacy and comparison with gefitinib

In our study, gefitinib served as a positive control and demonstrated significant antitumor efficacy. Notably, Wen et al. recently reported that circSETD3 promotes gefitinib resistance in NSCLC cells through the FXR1/ECT2 pathway, which inhibits JNK/Bim-mediated apoptosis (Wen et al., 2023). While their study focuses on non-coding RNA-mediated resistance mechanisms, our findings highlight metabolic reprogramming as an additional axis potentially contributing to therapeutic response. Zheng et al. further demonstrated that mitochondrial cristae remodeling and metabolic reprogramming—specifically through Dapk2 dysfunction and Mic60 lactylation—contribute to EGFR-TKI resistance and metastasis in lung cancer (Zheng et al., 2025). These findings collectively suggest that mitochondrial metabolic adaptation represents a convergent mechanism underlying both resistance to targeted therapies and the antitumor activity of metabolic modulators such as TPL. These complementary insights suggest that both RNA-based regulatory networks and metabolic adaptations may jointly influence gefitinib sensitivity.

Consistent with broader patterns of kinase dysregulation in NSCLC (Pérez et al.) and the emerging role of adaptive vesicular responses to targeted agents (Gladkiy et al.) (Pérez et al., 2024; Gladkiy et al., 2025), our findings support the concept that multi-target strategies—such as TPL—may overcome resistance by simultaneously interfering with signaling and adaptive survival pathways.

### 4.4 Cell line-specific responses and limitations

The observed discrepancies between H1299 and NCI-H460 cells may reflect inherent differences in their genetic backgrounds or metabolic dependencies. For instance, NCI-H460 cells are derived from a large cell lung carcinoma, while H1299 cells originate from a lymph node metastasis of a non-small cell lung adenocarcinoma (Sellers et al., 2019; Lim et al., 2018). These distinct origins likely contribute to differences in their genetic mutations, signaling pathways, and metabolic profiles. Specifically, NCI-H460 cells are known to harbor mutations in the KRAS and TP53 genes, which are associated with altered metabolic pathways such as glycolysis and glutaminolysis. In contrast, H1299 cells exhibit a deletion in the TP53 gene and may rely more heavily on alternative metabolic pathways, such as fatty acid oxidation or nucleotide synthesis, to sustain their growth and survival.

Additionally, previous studies have highlighted the metabolic heterogeneity of NSCLC cells, with different cell lines exhibiting varying dependencies on glucose, glutamine, and other nutrients (Wu et al., 2021). For example, some NSCLC cell lines show a strong reliance on glycolysis (the Warburg effect), while others preferentially utilize oxidative phosphorylation or other metabolic pathways (Mendes et al., 2023). These differences may explain why TPL treatment elicited more pronounced metabolic changes in NCI-H460 cells compared to H1299 cells. Further studies are needed to elucidate the underlying mechanisms driving these cell line-specific responses, such as transcriptomic or proteomic profiling to identify key regulatory genes and proteins. Moreover, the tumor microenvironment (TME) *in vivo*, which includes stromal cells, immune cells, and extracellular matrix components, can significantly influence tumor metabolism and drug responses. Since cell line models lack the complexity of the TME, future investigations using patient-derived xenografts (PDX) or 3D co-culture systems may provide more clinically relevant insights into the metabolic effects of TPL. Understanding these cell line-specific differences is crucial for determining the generalizability of our findings and for developing personalized therapeutic strategies targeting the metabolic vulnerabilities of NSCLC.

**FIGURE 9:**
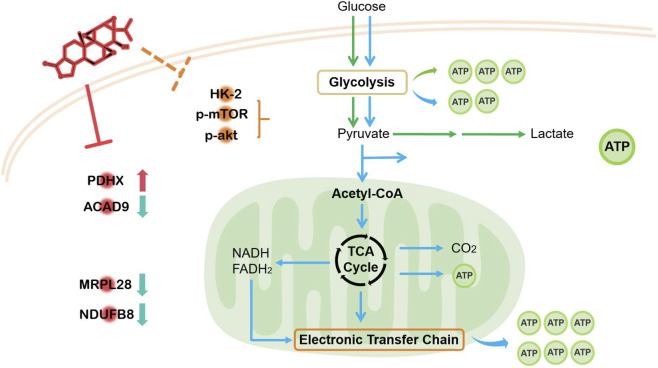
Proposed mechanism of action of TPL in targeting metabolic reprogramming to inhibit NSCLC progression.

In summary, this study provides the first evidence that triptolide reprograms NSCLC metabolism through a mechanism involving glycolysis suppression (*via* HK2 downregulation) and alterations in mitochondrial pyruvate oxidation associated with PDHX changes (Figure 9). These effects are accompanied by ROS accumulation, mitochondrial membrane depolarization, and impaired energy metabolism, ultimately leading to growth arrest and apoptosis. The *in vivo* efficacy of TPL in NSCLC xenografts further supports its therapeutic potential. Together, our findings suggest that TPL warrants further investigation as a potential metabolic modulator in NSCLC.

## 5 Conclusion

Triptolide (TPL) exerts antitumor effects in non-small cell lung cancer (NSCLC) by reprogramming metabolism, with PDHX identified as a key node in this metabolic disruption. TPL suppresses glycolysis while forcing a compensatory but unsustainable reliance on oxidative phosphorylation, ultimately leading to mitochondrial dysfunction and ATP depletion, accompanied by ROS accumulation, which collectively inhibit NSCLC growth. Metabolomics and DARTS proteomics identified key metabolic changes and potential interactions between TPL and PDHX, highlighting its role in energy metabolism. *In vivo*, TPL significantly reduced tumor growth in a dose-dependent manner with no overt signs of toxicity observed under the current experimental conditions, warranting further investigation with comprehensive safety and pharmacokinetic assessments. These findings suggest TPL as a potential therapeutic agent for NSCLC, warranting further clinical investigation.

## Data Availability

The datasets presented in this study can be found in online repositories. The names of the repository/repositories and accession number(s) can be found in the article/Supplementary Material.
