# Supplementary material for: Integrating multi-omics approaches reveals metabolic reprogramming and identifies PDHX as a candidate node in triptolide-treated non-small Cell lung cancer
Source: Front Pharmacol. 2026 Apr 21;17:1785207. doi: 10.3389/fphar.2026.1785207 (PMC13139181; doi:10.3389/fphar.2026.1785207)
Supplement: Supplementary file 1 [file DataSheet1.pdf]

Supplementary Material

1 Supplementary Data

Supplementary Material should be uploaded separately on submission. Please include any supplementary data, figures and/or tables.

Supplementary material is not typeset so please ensure that all information is clearly presented, the appropriate caption is included in the file and not in the manuscript, and that the style conforms to the rest of the article.

2 Supplementary Figures and Tables

2.1 Supplementary Figures

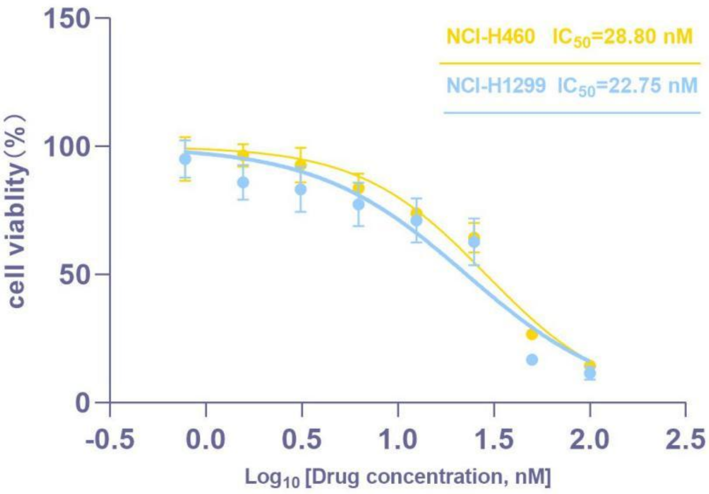

Supplementary Figure 1. NCI-H1299 and NCI-H460 cells treated with 0 – 100 nM TPL

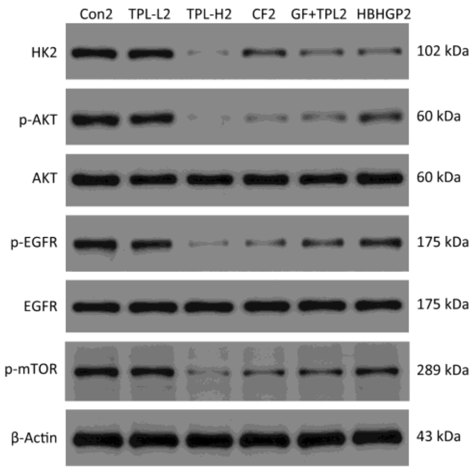

Supplementary Figure2. Full unedited western blot 1

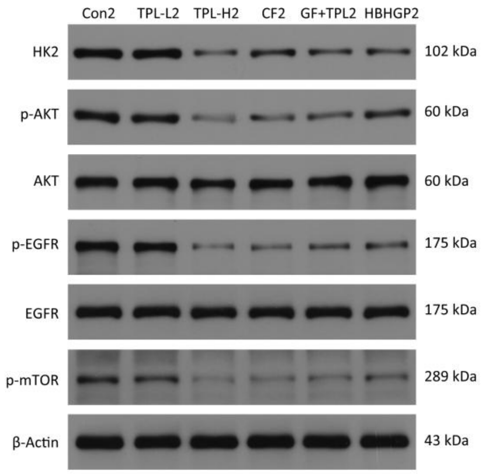

Supplementary Figure3. Full unedited western blot 2

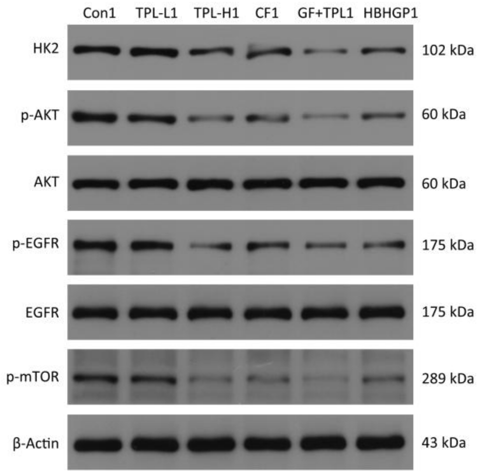

Supplementary Figure3. Full unedited western blot 3

2.2 Supplementary Table

Supplementary Table 1 Potential energy metabolism-related proteins directly bound to TPL detected by the DARTS test

Accession	Gene Symbol	MW [kDa]	calc. pI	drug_VS_con_Ratio	drug_VS_con_Pvalue	Mean_drug	Mean_con
O00330	PDHX	54.1	8.66	1.260555905	0.016303892	203836.3281	161703.5208
P11216	PYGB	96.6	6.86	0.823320239	0.039855866	3817357.44	4636540.268

P04183	TK1	25.5	8.51	0.81587216	0.019793866	271369.7292	332613.0521
Q96G23	CERS2	44.8	8.98	0.805978454	0.043904798	389444.1042	483194.1875
P12277	CKB	42.6	5.59	0.64068263	0.016669827	6543659.211	10213573.62
O95169	NDUFB8	21.8	6.8	0.020835219	0.000337741	2125.670654	102022.9557
